# Formative research for the development of baby water, sanitation, and hygiene interventions for young children in the Democratic Republic of the Congo (REDUCE program)

**DOI:** 10.1186/s12889-021-10246-5

**Published:** 2021-03-01

**Authors:** Jennifer Kuhl, Lucien Bisimwa, Elizabeth D. Thomas, Camille Williams, Joseph Ntakirutimana, Nicole Coglianese, Sarah Bauler, Ruthly François, Presence Sanvura, Jean Claude Bisimwa, Patrick Mirindi, Christine Marie George

**Affiliations:** 1 Department of International Health, Program in Global Disease Epidemiology and Control, Johns Hopkins Bloomberg School of Public Health, 615 N. Wolfe Street, Room E5535, Baltimore, MD 21205-2103 USA; 2 Food for the Hungry, Phoenix, USA

**Keywords:** Democratic Republic of the Congo, Qualitative methods, Water, sanitation, and hygiene, Care groups, Infants

## Abstract

**Background:**

Research exploring the unique exposure pathways to fecal pathogens for young children and innovative water, sanitation, and hygiene (WASH) interventions for susceptible pediatric populations is needed to reduce the burden of diarrheal diseases and stunting globally. The Reducing Enteropathy, Diarrhea, Undernutrition, and Contamination in the Environment (REDUCE) program seeks to 1) identify exposure pathways to fecal pathogens that are significant contributors to morbidity for young children in South Kivu, Democratic Republic of the Congo, and 2) develop and evaluate scalable interventions that reduce fecal contamination and exposure from these pathways. The formative research portion of the project sought to identify feasible and acceptable WASH interventions to modify behaviors found to be associated with diarrheal disease and impaired growth in our REDUCE cohort study.

**Methods:**

Ninety-one semi-structured interviews, 6 focus group discussions, and a pilot study of 102 households were conducted during 24 months of formative research. Thirty-one interviews and six focus group discussions were conducted with caregivers, community health workers, and village leaders to explore existing WASH practices and to identify barriers and facilitators to WASH behaviors. Findings were organized using the Integrated Behavioral Model for Water, Sanitation and Hygiene to facilitate interpretation and identify determinants to Baby WASH behaviors in this setting. Care Group modules and enabling technology were developed based on exploratory findings and then revised during a two-part, iterative pilot study. Sixty interviews were conducted with participants in a pilot study of the REDUCE Baby WASH Care Group modules to learn about their experiences with the intervention.

**Results:**

Six REDUCE Baby WASH Care Group modules were developed based on formative research findings and covered the following topics: 1) living with animals; 2) child mouthing of fomites and feces; 3) composting animal feces; 4) child feces disposal; 5) handwashing with soap; and 6) water treatment.

**Conclusion:**

This study took a theory-driven and evidence-based approach to formative research and the development of the REDUCE Baby WASH Care Group modules. Intervention design focused on interrupting the exposure routes for infants and young children to fecal pathogens in the environment and promoting low-cost, low-burden Baby WASH behavioral recommendations and enabling technology. These developed REDUCE Baby WASH Care Group modules are currently being rolled out to over 1,000,000 beneficiaries in Democratic Republic of the Congo.

**Supplementary Information:**

The online version contains supplementary material available at 10.1186/s12889-021-10246-5.

## Background

Despite advances in health and sanitation worldwide, diarrheal disease is still a leading cause of death among children under 5 years globally [1]. Diarrhea and malnutrition can be cyclic in their impact, as undernourished children are more susceptible to infections and children suffering from diarrhea absorb fewer nutrients, exacerbating malnutrition [2]. Furthermore, recent research suggests that even if enteric pathogens are not causing symptomatic illness, carriage of these pathogens may contribute to environmental enteropathy, a condition that can reduce nutrient absorption in young children [3, 4].

In the recent decade, diarrheal diseases were responsible for approximately 10% of annual deaths among children under 5 years in the Democratic Republic of the Congo (DRC) [5]. In addition, 40% of children under 5 years in DRC are estimated to be stunted, with that number rising to 47% in rural areas. South Kivu province has the highest percentage of stunting in the country, with 53% of children being stunted [6]. Interventions to reduce childhood diarrheal disease, enteric infections, and stunting in rural areas of DRC like South Kivu are urgently needed.

Recent large-scale water, sanitation, and hygiene (WASH) intervention trials focused on traditional “F-diagram” fecal exposure pathways (fluids, fields, fingers, flies, food) have not shown expected improvements in diarrheal disease, environmental enteropathy, and child growth [7–9]. This is likely because interventions focused on traditional fecal exposure pathways do not adequately address exposure routes for infants and young children, such as child mouthing behaviors, contact with animals, and fecal contamination on fomites [10–14].

### REDUCE program

The Reducing Enteropathy, Diarrhea, Undernutrition, and Contamination in the Environment (REDUCE) program seeks to identify exposure pathways to fecal pathogens that are significant contributors to morbidity for young children in South Kivu, DRC, and to develop and evaluate scalable interventions that reduce fecal contamination from these pathways. The REDUCE program was conducted within the scope of a USAID/Bureau for Humanitarian Assistance (BHA)-funded Development Food Security Activity (DFSA). Globally, FFP-funded DFSAs often work with rural, ‘last mile’ and ultra-poor communities to improve food and nutrition security outcomes. Our recent REDUCE program cohort study in South Kivu found that child mouthing of feces and soil, touching rabbits and guinea pigs, and the presence of feces in the child’s sleeping space were associated with diarrhea and stunting among young children [15]. These findings highlight the need for further research exploring the unique exposure pathways to fecal pathogens for young children and innovative WASH interventions to disrupt them.

### Care Group model

The Care Group model is a form of peer-to-peer education and support developed in the late 1990s by child survival staff at World Relief [16–18]. The model relies on existing community relationships to disseminate information as a means for health and nutrition communication programs to inexpensively go to scale. In the Care Group model, one paid educator, called a “Promotor,” selects approximately 10 Care Group Volunteers who live within a given area to provide communication modules to a group of neighboring households. Every 2–4 weeks, the Promotor convenes a meeting with the Care Group Volunteers and teaches them a new communication module containing 1–3 health-related key health protective behavioral recommendations. After meeting with the Promotor, the Care Group Volunteers return to their own communities and share module behavioral recommendations and other content with neighboring households during household visits. Each Promoter is able to train several groups of Care Group Volunteers, and each Care Group Volunteer is responsible for approximately 10 households; thus, a single paid Promotor can reach between 400 and 1000 households every month. The Care Group model has been implemented in 27 countries and has been shown to increase coverage of child survival programs and reduce under five mortality rates [19]. The Care Group approach presents a promising strategy for delivery of WASH interventions for young children (Baby WASH) to the caregivers of young children in rural, underserved communities.

### Study objectives

This paper presents the formative research conducted to develop feasible and acceptable Baby WASH interventions for young children for delivery by the Care Group model. The goals of the formative research were: (1) to identify the barriers and facilitators for caregivers of young children to perform target WASH behaviors; (2) to develop evidence-based and theory-driven REDUCE Baby WASH Care Group modules to reduce exposure to fecal pathogens among young children; and (3) to assess the acceptability and feasibility of delivering the developed modules.

### Project overview

Formative research activities included two components: (1) exploratory research and (2) intervention development and piloting. In the first component, we conducted semi-structured interviews and focus group discussions to explore existing WASH practices and to obtain recommendations for intervention development (guides are available in Supplementary File 1). In the second component, based on exploratory formative research findings, we developed Care Group modules and identified candidate enabling technologies to facilitate Baby WASH behavioral recommendations. We then conducted two rounds of intervention piloting to assess the acceptability and feasibility of the behavioral recommendations and enabling technologies developed for each module.

The processes and results for each component are presented by study component below. For each component, this manuscript presents the findings related to child mouthing behaviors, living with animals, and child feces disposal and the related Care Group modules, as these were the focus of the REDUCE program and identified as sources of fecal pathogen exposure associated with impaired growth and diarrheal disease in this setting [15]. Formative research findings and the related Care Group modules for handwashing and water treatment behaviors are available in Supplementary File 2.

All research activities were conducted in Walungu Territory in the Province of South Kivu in DRC.

## Component I: Exploratory research 

### Methods

Between October 2017 and December 2018, 31 semi-structured interviews and six focus group discussions were conducted with mothers, fathers, and other caregivers of young children, community health workers, and community leaders. To participate in this first component of the study, individuals needed to reside in Walungu Territory and be 12 years of age or older. Formative research activities were guided by the multi-level Integrated Behavioral Model for Water, Sanitation and Hygiene (IBM-WASH), which is based on health behavior change being influenced by various dimensions (contextual factors, psychosocial factors, technological factors) at multiple levels (structural, community, interpersonal, individual, habitual) [20]. Activities were designed to explore the influences of health behavior across these factors and levels.

Trained Congolese staff conducted interviews and moderated focus group discussions in Swahili, using semi-structured guides to facilitate discussion. In an iterative process, interview and focus group discussion guides were expanded and revised as saturation was reached in some topic areas and early findings suggested new areas of inquiry. Topics explored during interviews and focus group discussions focused on child health and potential fecal exposure routes for infants and young children in the domestic environment in this setting, including: family roles and responsibilities, child care practices, child mouthing behaviors, child feces disposal, family interactions with animals, handwashing and other hygiene practices, water treatment practices, and frequency and understanding of common illnesses.

Research staff completed comprehensive French debriefs of the interviews and focus group discussions directly following the activities to facilitate rapid analysis of emergent findings and guide future iterations of data collection. Focus group discussion debriefs were augmented with summaries based on written notes taken in French during the discussions by a designated note taker. Semi-structured interviews and focus group discussions were transcribed from recordings in Swahili and then translated into French by the research team.

French transcriptions, debriefs, and summary notes were coded in ATLAS.ti 8 qualitative data analysis software. A preliminary codebook was developed based on research questions. The codebook was revised and expanded after coding initial interviews for emergent themes. Findings were then organized using IBM-WASH to facilitate interpretation [20].

## Results

### Child mouthing behaviors

We found that when parents work outside of the home, most often as farmers, infants and toddlers are often left with older siblings or are left alone, making it difficult for parents to monitor mouthing behaviors of young children.

*“I leave [the baby] with his four-year-old brother. I leave them and I say ‘I'm leaving you here and I'll come back. Rest and play until the others get back from school.’" –Mother*

Caregivers said that most children play in the yard on the bare earth, and that very young children put “whatever they find” in their mouths, including soil, dirty objects, and feces because they “do not know any better.” Caregivers described a connection between mouthing or eating dirt or dirty things and *nyoka mtumbu*, or ‘snakes in the belly,’ a local term for intestinal worms.

Caregivers expressed concern that if their child became ill from this mouthing behavior, they would not have the financial resources to pay for treatment.

*“Sometimes a child can get sick and you leave him at home because you lack the money to treat him… They're not going to give you medicine for nothing. And if you treat him today, tomorrow you can't [get treatment] because you won’t have money.”–Mother*

When asked what kinds of games children regularly play, participants said children pretend to make “fufu” (a local staple food made of corn or cassava) using mud, and that while older children know better than to eat “mud fufu,” they may feed it to younger siblings, who swallow it.

When asked by interviewers whether young children ever ate feces, or put feces in their mouths, participants voiced disgust at the idea and said that feces contain “microbes,” a term frequently used to describe germs.

*“[Feces is] poison – that's what our parents used to say. A person can't eat feces, and if he eats it, he won’t go on living because it contains a lot of microbes.”–Community leader*

Several participants suggested a carpet or a mat to prevent children from touching or playing with dirty things.

*“You see here, outside, there is the ground, and if there was a clean place ... he wouldn't eat dirt. If we had a tarp, we could sit the child on the ground, and he wouldn't eat dirt anymore.”–Community leader*

Limited financial resources were reported as playing a major role in household decision-making and ability to purchase or construct clean and engaging spaces for children to play. When we asked about playmats as a possible intervention, some said a child might be happier to stay on a playmat if they were given toys to occupy them, but also noted that toys could be expensive.

*“If we were in a place with means, if we could buy the child a toy … that would help him not to touch everything.”–Community health worker*

Participants commented that children might not like a playmat if they were left alone on it but suggested that if other family members kept the child company, they would accept being placed on the playmat.

*“If a parent is present or other children with whom he plays, the child will not be afraid [of the playmat]”–Mother*

### Child feces disposal

Caregivers reported that young children defecate in their diapers, which are made from pieces of cloth (washed and reused) tied around the child’s waist, or that they defecate wherever they are sitting in the yard. While a few participants mentioned digging a hole to bury children’s feces, most reported using a hoe to remove feces from the yard and disposing of it in the latrine. Participants reported a past practice of digging a small hole beside the latrine for children to use for defecation (children’s toilet) but noted that this practice was later discouraged by health educators who said the holes were not deep enough to prevent contamination.

*“We had a small toilet for children but people told us never to use these toilets again because they are a source of flies that bring germs everywhere in the neighborhood.” –Mother*

Participants noted that there might be some delay in disposal of child feces if parents were working away from the home, especially if the person looking after the child in their absence was preoccupied with other activities.

Participants described all human feces, including children’s feces, as contaminated with microbes and potentially dangerous to health. However, the feces of breastfed infants were seen by some as having fewer microbes than the feces of children who were already eating solid foods.

*“The feces of a small child who has already started to eat has many microbes, and an infant who has not yet started to eat all sorts of food does not have too many microbes.” –Mother*

When children defecated in diapers, participants said they washed the diaper and the child upon noticing the child needed to be changed. Most reported disposing of feces in the latrine; however, some reported disposing of some diaper wash water in the yard or field.

*“I throw [the feces] in [the toilet] after I return. I wash his clothes and spread them out… I throw the first wash water (used for washing diapers) in the hole (latrine) and the second wash water I throw in the yard.” –Grandmother*

### Living and interacting with animals

Participants reported that their communities had a long history of living with animals of all varieties, including cows, pigs, goats, rabbits, guinea pigs, and chickens. As a long-standing way of life, raising animals was not seen by most as a source of concern or illness.

*“We've lived together with animals for a long time, that's how we raise them here. We're already used to [living with animals] and it doesn't cause a problem.” –Community leader*

Animals were seen as an important source of income and food. Many families kept small animals, such as rabbits and guinea pigs, in their kitchen areas, and some families kept larger animals, such as cows and pigs, in their kitchens and homes at night to prevent them from being stolen.

*“It's also to avoid theft. When you have two or three houses, you will put the animals you're raising in one house and you will stay in another. Two or three days later, people will realize that nobody is sleeping in the house with the animals, and then thieves will visit you and steal [them]. Like me – yesterday I had a chicken stolen from the house because they knew no one was spending the night [in the same room as the chicken].” –Community leader*

Some participants said that they would prefer to keep their animals in a separate home or structure; however, many among this group noted that it was beyond the means of many families to construct a separate space for animals. Commenting on the practice of sharing a home with animals, a few participants felt animals should not sleep in the same place as people.

*“An animal can’t spend the night in the same place as a person. He must have his own house …but sharing the house with the animal? No, because the animal has a lot of illnesses.”–Father*

Some respondents also expressed concerns that animals living in close proximity with humans might cause the animals to be hurt by children playing.

There were differing perspectives on the safety of animal feces. Some participants said that likeall feces, animal feces was dirty, had microbes, and could cause illness. Others viewed some animal feces, such cow dung, as relatively clean.

*“After touching [cow] dung we always say, ‘It's not dirty,’ because it has come freshly from the stomach of a cow where there is no dirt.”–Father*

When participants were asked if they would be open to sweeping up animal feces and throwing it in the latrine, participants voiced very strong opposition and argued for its utility in agriculture.

*“Animals’ excrement is important to us because we use it as fertilizer.”–Father*

Participants frequently mentioned that small animals, such as rabbits and guinea pigs, could not be kept outdoors because if small animals were allowed to get cold, they would die.

*“The kitchen is the best place [for rabbits and guinea pigs] … if we put them [outside, it] is very cold and they will die. I once kept guinea pigs [outdoors] and they died – and I even put the rabbits [outside], and they died too, and people began to advise [me] not to keep animals where there is no fire, so I tried putting them in the kitchen and they did not die anymore.” –Mother*

### Cross cutting themes: limited resources and competing priorities

Two major interconnected themes emerged in the exploratory research: limited resources and competing priorities. Regarding limited resources, many participants said that they did not have “the means” to purchase various materials necessary to practice positive WASH behaviors, such as soap for handwashing, a playmat for children, and supplies to construct a hutch for small animals.

*“People spend the night there [in the same house with animals] because they lack the means … we don’t have the means to live in one house and have another house for the animals.” –Father*

With respect to competing priorities, this was expressed as relating to both time and to the already limited resources. For example, many participants felt they could not supervise children because they needed to work in the field.

*“This is the problem we have with keeping a child's hands clean—we can't stay with him all the time …You will go to the field and leave the child to eat and the child will rush to eat without washing his hands.” –Community leader*

This arose in relation to other chores with parents stating, for example, that they might not have time to boil water because they needed to make dinner. They also noted that material items like soap and water might not be available for handwashing or diaper cleaning because they had instead been used for washing dishes or bathing.

## Component II – Intervention development and piloting

### Methods

The selection of topic areas for the REDUCE Baby WASH Care Group modules was informed by findings from our REDUCE prospective cohort study on fecal exposure pathways for infants and young children [21–23]. Topic selection was further refined based on the themes that emerged from Component I findings. The five broad topic areas selected included: child mouthing, animal interactions, child feces disposal, handwashing with soap, and water treatment. Target behaviors within the five topic areas were identified through quantitative analysis and psychosocial factor analysis [15].

Findings from Component I were then organized by topic area into IBM-WASH matrices (tables) to help identify factors likely to affect WASH-related behaviors in this setting. We then took guidance from completed tables to identify behavioral recommendations, visual aids, communication materials, and enabling technology to change or reinforce target behaviors. Based on this, five intervention modules were developed: (1) Protecting Children from Dangers in the Dirt, (2) Hutches for Small Animals, (3) Safe Child Feces Disposal, (4) Handwashing with Soap, and (5) Water Treatment. Development and content for the Handwashing with Soap and Water Treatment modules are outlined in Supplementary File 2.

In Tables 1, 2, 3, and 4 we present findings from Component I and II (discussed below), organized by IBM-WASH dimension, to demonstrate how the important contextual, psychosocial, and technological factors identified through exploratory research informed content for each module [24]. By organizing our findings this way, we were able to consider the multi-level and multi-dimensional factors influencing our target WASH behaviors. In Supplementary File 3, we provide quotes from interviews and focus group discussions to support findings.

**Table 1:** Design of the Protecting Children from Dangers in the Dirt Care Group Module and Enabling Technology, Organized by IBM-WASH Dimension

Dimension of IBM-WASH	Implications for Intervention Design
*Contextual dimension*	
Physical environment: Outdoor and indoor spaces are soil/made from soil, making it difficult to separate children from dirt during play.	Playmats selected as an enabling technology to reduce children’s time spent in direct contact with soil.
Roles and responsibilities: Because adult caregivers often work away from the home, infants and toddlers may be left in the care of school-age children (4–12 years).	Songs describing the danger of mouthing dirty things developed to make behavioral recommendations about keeping young children on playmats accessible to school-age children.
Play between children: Children commonly play with “mud fufu” with each other, and younger children sometimes eat it.	Narrative illustration at the beginning of the module showing an older child making mud fufu and a younger child eating it, and then showing the younger child becoming ill.
*Psychosocial dimension*	
Dislike/perceived threat: Caregivers dislike when children mouth soil or dirty things because they will become ill or get “snakes in the belly”.	Pictorial instructions included in module describe the connection between mouthing dirty things and developing intestinal worms and/or diarrhea.
Concern/fear: There is a concern that small children will be upset/afraid/feel trapped if they are left alone on a mat or in a play yard to play.	Selection of playmats over play yards as an enabling technology to reduce concern/fears of child feeling trapped or scared. Encouragement of caregivers to play with children on the playmat, making the playmat a happy place for children and families.
Cost: There is a high perceived cost associated with children getting diarrhea.	Narrative illustration included in module describes the financial burden associated with a child who becomes ill from eating dirt.
Existing habits: Children play with whatever they find in the yard; Caregivers are accustomed to putting children on the ground when they are busy with other activities.	Pictorial instructions included in module show how to build and provide children with safe toys made from local materials. Encouragement of caregivers to always place children on the playmat, inside or outside, to encourage habit formation.
* Technological dimension*	
Manufacturing/access: Commercial playmats and play yards are not manufactured in DRC, and not distributed to markets accessible to rural populations.	Candidate playmats identified from locally available, low-cost, already in-use materials.
Strengths/weaknesses of hardware: Some caregivers prefer a plastic flooring playmat, because it is larger, more durable, and easier to clean than a rice bag.	Plastic flooring playmat selected as final enabling technology.

**Table 2:** Design of the Safe Disposal of Child Feces Care Group Module and Enabling Technology, Organized by IBM-WASH Dimension

Dimension of IBM-WASH	Implications for Intervention Design
*Contextual dimension*	
Access: Most households have their own pit latrine, which may facilitate disposal of child feces in the latrine.	Encouragement to always dispose of child feces in the latrine, either using a hoe or leaves. Recommendation to bury feces if latrine not available.
Roles and responsibilities: Because caregivers are busy with other tasks or away from the home, there may be a delay in removing feces from the yard.	Pictorial instructions included in module recommending the adult caregiver (e.g. mother) to dispose of all child feces from the yard as soon as they return to the home.
*Psychosocial dimension*	
Beliefs: Caregivers believe that feces of a breastfed infant have fewer microbes than the feces of a child who is eating solid food.	Narrative illustration included in module describes how an infant’s feces can carry microbes, and how other family members can become ill from these microbes.
Disgust and dirt reactivity: Caregivers have a disgust reaction to child feces, consider them dirty, and want to remove them from the yard.	Reinforcement of these feelings by showing illustrations of intestinal worms in feces in the module.
Existing habits: Caregivers accustomed to using a shovel or hoe to remove feces from the yard; Caregivers previously accustomed to digging small holes beside the latrine for young children to defecate in; Child potties not in-use, but cloth diapers common; Some caregivers throw water from cleaning diapers in yard.	Hoe promoted as the enabling technology to remove child feces and dispose of it into the latrine. Leaves promoted to remove feces if hoe not available. Pictorial instructions included in module reinforcing removal of child feces with hoe, plus instructions for cleaning hoe with detergent after disposal. Narrative illustration included in module describes the dangers of throwing water with feces in the yard. Recommendation that water from cleaning feces or diapers be thrown in the latrine (or buried).
* Technological dimension*	
Manufacturing/access: Child potties not available in markets accessible to rural populations.	Child potties not included as a candidate enabling technology.
Convenience: Households typically have a hoe; households typically have a pit latrine.	Hoe promoted as the enabling technology to remove child feces and dispose into the pit latrine.

**Table 3:** Design of the Living with Small Animals Care Group Module and Enabling Technology, Organized by IBM-WASH Dimension

Dimension of IBM-WASH	Implications for Intervention Design
*Contextual dimension*	
Climate: Small animals (e.g. rabbits and guinea pigs) cannot be kept outside due to weather.	Hutches for small animals selected as an enabling technology to facilitate keeping animals close to home but separate from children’s playing/eating/sleeping spaces (i.e. hutch in or attached to kitchen).
Theft: Theft of domestic animals is possible if they are left in the yard at night, contributing to the practice of keeping animals inside alongside humans (e.g. in the kitchen or bedroom).	
Roles and responsibilities: Caregivers and other adults are away from home during the day, leaving animal feces around the home untended to throughout the day.	Pictorial instructions included in module recommend adults clean the compound daily (by sweeping) and clean the animal hutch a few times per week. Addition of a Composting Module.
Household resources: Preference for keeping animals in a separate structure limited by household resources (e.g. space, finances).	Candidate animal hutches constructed from low-cost, locally available materials.
Household income and food security: Animals provide an important source of nutrients and potential cash flow.	Narrative illustration included in module acknowledges the importance of animals and highlights the importance of keeping them safe by giving them a home (i.e. hutch).
*Psychosocial dimension*	
Cultural identity: There is a long history of living with animals in this community. Animals provide food and economic support.	
Knowledge/perceived threat: Some animal feces are considered dirty and/or harmful for human health, others are not.	Narrative illustration included in module describes that all animals can carry germs that can make household members ill.
Safety concerns: Concern that children will hurt animals by playing with them roughly if they are kept in close proximity to each other.	Narrative illustration included in module emphasizes the value of building a safe space for animals.
Existing habits: Very few households keep guinea pigs in hutches, while some do keep rabbits in hutches.	Information included in the module about safely keeping guinea pigs in a contained space.
* Technological dimension*	
Manufacturing/access: Small, inexpensive animal hutches accessible to rural populations.	Candidate animal hutch design able to be built by household members from low-cost, locally available materials, used by some households currently.

**Table 4:** Design of the Composting Care Group Module and Enabling Technology, Organized by IBM-WASH Dimension

Dimension of IBM-WASH	Implications for Intervention Design
*Contextual dimension*	
Theft: Theft of animal feces is possible, given their value for fertilizer. Some households keep feces in bags inside the kitchen or living space to avoid theft.	Pictorial instructions included in module recommend creation of a compost heap far from where children play.
Livelihood/crop productivity: Animal feces are highly valued as agricultural fertilizer, resulting in opposition to dispose of animal feces in the latrine.	Narrative illustration included in module emphasizes the benefits of composting animal feces for generating fertilizer.
*Psychosocial dimension*	
Existing habits: Some households make compost for fertilizer from animal feces and/or food waste; most households put animal feces directly into the field after sweeping/cleaning to remove feces from the living area.	Pictorial instructions included in module recommend that feces be put in a compost pile with other household waste.
* Technological dimension*	
Manufacturing/access: Small, inexpensive compost piles accessible to rural populations.	Candidate compost pile able to be built by household members from low-cost, locally available materials, used by some households currently.

Between January 2019 and December 2019, we conducted a two-phase pilot to explore the acceptability and feasibility of the behavioral recommendations and the enabling technology for each module. To participate in the pilot study, individuals needed to reside in Walungu Territory and have a child under 5 years in their household.

#### Phase I

Phase I focused on exploring acceptability and feasibility of enabling technology as well as pre-testing flipbooks with intervention content for each module.Thirty households across five villages participated in Phase I; each household received only one intervention module, and this differed from the interventions received by their neighbors.

##### Child mouthing behavior

Based on statements made by caregivers that they would value being able to place their child on a mat, two options for playmats were tested in Phase I of the pilot: a playmat made from a large rice bag and a playmat made from a piece of plastic flooring. Both options were locally sourced and already being used by some households within the program area. The rice bag playmat cost considerably less than the plastic playmat (0.50 vs. 3 USD). A play yard was also considered; however, because of the high cost (80 USD) and caregiver concerns that the child would feel trapped, this item was not included in Phase I piloting.

The expressed relationship between mouthing dirt or dirty things and “snakes in the belly” (intestinal worms) led to the inclusion of a pictorial depiction of this disease transmission route in the flipbook for the Protecting Children from Dangers in the Dirt Module. The flipbook also explained the risk of young children getting intestinal worms from eating mud fufu.

##### Living and interacting with animals

During exploratory research, we observed rabbits and guinea pigs in kitchens running over plates and defecating on plates, utensils, and other dishes. During this time, recommendations were made to have a separate space for housing animals. Animal hutches were observed in the kitchen and sleeping spaces in some households already, so we decided to introduce an animal hutch made of locally available materials for housing rabbits, guinea pigs, ducks, and chickens in Phase I piloting. The objective of these hutches was to reduce human contact with animals in indoor living spaces in the home. Participants voiced strong resistance to building hutches outside due to concerns that animals would be stolen or would die from the cold, so hutches were built indoors. The design for hutches was based on the ones observed in the community and had three levels. The top level was for rabbits, the second for guinea pigs, and the bottom level was for chickens, turkeys, and ducks. Rabbits and guinea pigs stayed in the hutch throughout the day and night. Hutches were constructed from rags, wood, and bamboo.

Constructing separate pens or houses for larger animals was not possible due to cost and property space constraints. In addition, larger animals often spend time away from the home during the day grazing for food, and therefore defecate in the household compound less often than animals that are consistently on the household compound. Participants considered a holding pen for chickens as undesirable because cooped chickens would need to be fed, whereas free-range chickens feed themselves. For these reasons, chickens were only kept in the animal hutches at night on the bottom level.

Sweeping up animal feces from the household compound and disposing of it far from where children play was recommended by participants as the preferred method of managing fecal contamination from animal feces on the household compound. The flipbook designed to accompany the hutches emphasized worms and germs present in animal feces and outlined the importance of sweeping up any animal feces seen on the household compound daily, and cleaning the animal hutch a few times per week. In addition, the module described that hutches would also keep animals safe, reflecting participant statements about the importance of animals to their families.

*Child feces disposal*  No new technology was introduced for the Safe Child Feces Disposal module, but households were requested to dispose of children’s feces and diaper wash water in the latrine. The module emphasized health risks, and possible economic risks, posed by all feces, including the feces of infants who were not yet eating solid food.

*Cross cutting themes* In consideration of concerns raised about limited means, all intervention technologies were low-cost or free and locally sourced. To address time constraints and competing priorities for caregivers, behavioral recommendations were included in the intervention only if they could be integrated into existing routines or required minimal extra work for caregivers.

Members of the research team introduced enabling technology and behavioral recommendations to caregivers of young children one-to-one with the aid of a picture-based flipbook; delivery took up to 45 min. Thirty households were asked to use the enabling technology and/or practice behavioral recommendations for up to 4 weeks. Twenty participants were interviewed about their experiences, and interviews were followed by comprehensive debriefs by the research team to facilitate rapid analysis and revision of intervention enabling technologies and flipbooks in preparation for Phase II of the pilot. The research team, which included local staff and an adult education expert, expanded each module to include additional visual aids and communication materials such as songs and then  revised them for delivery using the Care Group model.From this point, intervention modules will be referred to as the REDUCE Baby WASH Care Group modules to reflect this change.

#### Phase II

Seventy-two households in different villages from Phase I were selected for Phase II of the pilot. Based on findings from Phase I of piloting, described in the next section, a Composting Animal Feces module was added. All modules were introduced to 60 caregivers of young children using the Care Group model. The 60 households were assigned to 10 Baby WASH Care Groups, with 6 households per Care Group. Twelve households served as control households for the quantitative aspect of our pilot activities, which will be reported elsewhere (George et al. [15] in preparation).

Each REDUCE Baby WASH Care Group module was introduced using one initial group session and a second home visit. In the initial group session, the module’s behavioral recommendations were delivered using expanded picture-based flipbooks. In addition, the module’s corresponding enabling technology was built together by participants in one household of a Care Group participant. In the week following the group session, Care Group Promoters visited participating households to reinforce behavioral recommendations and investigate whether technology was being used as recommended. Care Group modules were reviewed with each pilot household, with sessions ranging from 30 min to 2 h in duration. Up to 6 months after receiving the modules, participating caregivers of young children were interviewed about their experiences and asked to suggest areas for improving module content and delivery. Forty-six follow-up interviews were conducted in Phase II, conducted by trained Congolese staff in Swahili. Research staff transcribed, translated and completed comprehensive debriefs of these interviews.

## Results

Results for both Phase I and Phase II of the pilot are presented by each REDUCE Baby WASH Care Group module developed.

### Protecting Children from Dangers in the Dirt Module

#### Pilot – Phase I

During Phase I follow-up interviews, caregivers reported using both the plastic and rice bag playmats frequently and without much difficulty, though some reported a preference for the plastic playmat because it was larger, more durable, and easier to clean.

Caregivers reported that playmats reduced mouthing of dirt by children.

*“Before finding this playmat, [the baby] often picked up dirt on the floor and ate it, but since I received this playmat, he no longer picks up the dirt.” – Mother*

Caregivers also said their family members and neighbors noticed that the playmat was preventing children from putting dirt in their mouths.

*“My husband really appreciates the playmat and has said that it protects the child from dirt.”–Mother*

Some caregivers felt that their child was healthier after using the playmat.

*“[The child] is there [on the playmat] so he no longer eats dirt. This is what I observed as a change, and his health improved” –Mother*

Parents also mentioned that they tried to act on what they had seen in the flipbook.

*“In relation to the messages in the flipbook concerning these photos, I also forced myself to do what I had seen through these photos.”–Mother*

### Intervention refinement for pilot – Phase II

Based on Phase I findings that the plastic playmat was considered more durable by participants than the rice bag, this playmat was selected for Phase II piloting. A narrative illustration featuring a child who becomes ill after putting dirt in his mouth was also added to introduce this module. The story describes the financial costs a family faces if the child falls ill. The finding that school-age children often care for infant and toddler siblings led to the development of songs describing the risks of mouthing dirty things, with the intention of making content accessible to children as well as adults. Finally, suggestions for safe, homemade toys, such as cloth dolls, were presented in the module to address caregivers’ concerns that they could not afford toys. Frequent cleaning of these toys with water and soap or detergent powder was recommended.

#### Pilot – Phase II

In Phase II follow-up interviews, caregivers continued to respond positively to the playmat. One caregiver mentioned bringing the playmat to the field with her.

*“Each day I used it all day, I would spread it out there in the field and the other kids would sit on it…or even when it's time to feed everyone, they sit there.”–Mother*

Caregivers reported cleaning the playmat when it was dirty, as they had been instructed to in the Care Group module. They also reported using the playmat while they were doing other chores.

*“I can be preparing a meal or doing laundry and I’ll spread [the playmat] out and sit him on it.”–Mother*

Caregivers also mentioned that the playmat prevented food given to young children from dropping on the ground.

*“I also liked it because there are times that the child can drop his food on the playmat and will take it back to eat it because there is no dirt. But if it falls on the ground, it becomes infected with microbes.”–Mother*

With the increased use of the playmats, some caregivers report that theirs tore. Many of these caregivers, however, said they were able to sew the playmats back together and continue using them.

### Safe Child Feces Disposal Module

#### Pilot – Phase I

The existing practice of removing child feces with a hoe and disposing of it in the latrine was considered a good option because most families owned a hoe. As such, no additional technology was introduced during Phase I piloting. The flipbook reiterated the importance of disposing of feces in the latrine, either using a hoe or leaves, and described the necessity of washing diapers thoroughly and then disposing of all diaper water in the latrine.

##### Intervention refinement for Phase II piloting

For Phase II piloting, behavioral recommendations and a narrative illustration were added to the Safe Child Feces Disposal Module. The module sought to reinforce that feces are harmful by showing pictures of “snakes in the belly” (intestinal worms) adjacent to pictures of feces. The module also illustrated the importance of throwing all diaper wash water into the latrine rather than in the field, and cleaning the hoe or tool used to transfer child feces to the toilet with ash, soap, or detergent powder. It also encouraged caregivers to remove feces from the yard immediately upon returning from an absence from home.

#### Pilot – Phase II

Caregivers reported understanding the module’s behavioral recommendations and were able to recall recommendations about the proper disposal of children’s feces.

*“I saw that it's good because there were so many mothers who removed [children’s] feces by throwing it in the cassava [fields] but according to the teachings you showed us, children’s feces belong in the toilet. I saw that these teachings were good and changed a lot of people.”–Mother*

Caregivers incorporated lessons regarding disposal of wastewater from cleaning child feces and diapers into their daily practices.

*“Before, when I finished washing diapers, I threw the first water in the toilet, and the rest in the yard. But your lessons told us that when we wash diapers, all the water must be brought to the toilet and that is what we do now.” –Mother*

Participants were also able to remember information given about cleaning the tools used for feces disposal.

*“I have a hoe, and before I would just throw the feces into the toilet, but your teachings showed me that when I’ve finished throwing away the feces, I should clean the hoe with soap or ashes.” –Mother*

### Hutches for Small Animals Module

#### Pilot – Phase I

Participants selected the location they wanted to put their animal hutches. Participants responded positively to the animal hutch module. Participants said that after the construction of the animal hutch, children no longer ate animal feces from the floor of the house.

*“The rabbit excrement, [the child] ate it but when [the rabbit] was placed in this [hutch], [the child] no longer does that.” –Mother*

Participants also said that the animal hutch kept animals from stealing food from household members.

*“[The rabbit] steals the children’s food… he goes up… and takes the children’s food and eats. He is a thief and when he is in this [hutch] he no longer steals.” –Mother*

Some participants reported using the animal feces from their hutch to directly fertilize their fields or keeping the feces in a bag or pile for a few days, and then using them for fertilizer.

*“I shake [the animal hutch] and then the feces falls to the ground. Then I take the broom and something on which I want to collect dirt, and then I store the [animal feces] in one place. After a few days I put in the field.”*–*Mother*

Participants reported fertilizing fields close to their house, where children would sometimes play and could eat the discarded animal feces.

*“I throw [the animal feces] in the onion field … my child eats it, what he sees is what he’ll eat …and [the field] is close.”–Mother*

### Intervention refinement for Phase II piloting

Given that participants who received animal hutches in Phase I responded to them positively, the same animal hutches were included in Phase II without additional modifications. Based on findings from the exploratory formative research and pilot Phase 1 feedback, we refined the Hutches for Small Animals Module. The introductory narrative illustration and behavioral recommendations throughout the module reiterated participants’ statements about the importance of animals to families and suggested that separating animals and children would keep both animals *and* children healthy. The narrative illustration and flipbook showed animal feces as a cause of disease among humans, and especially among young children. Based on the findings that families would not throw animal feces in a latrine and that families would put animal feces directly from their hutch into the field, it was decided that families should dispose of animal feces in a compost pit instead. This led to the development of the Composting Animal Feces Module.

#### Pilot – Phase II

Most pilot participants who received animal hutches in Phase II kept their guinea pigs and rabbits in them throughout the day and night, with some keeping their ducks, turkeys, and chickens inside at night. Animal hutches were constructed in the kitchen and sleeping areas of households. Animal hutches were not constructed outside of the house because of the expressed fear that animals would be stolen during the night. One participant was initially fearful that their animals would die in the hutch. However, after seeing that the animals did not die, the participant became more comfortable with using the hutch. As with Phase I, participants said they appreciated that, because of the hutch, guinea pigs no longer ran around the house. Participants also reported that having the animal hutches prevented young children from eating animal feces.

Participants noted that the hutches protected guinea pigs and rabbits from children hurting them and from being stepped on and prevented them from defecating throughout the house and eating human food in the kitchen.

*“[The children] played with [the guinea pigs] … they caught them all the time, but now [the children] don’t catch them anymore because [the hutch] was built for them. [When the children caught the guinea pigs] it wasn’t good because it happened that the [childern] killed [the guinea pigs].”–Mother*

*“I see that [the animal hutch] is good because guinea pigs do not roam around the house as they did before. There were times that the guinea pigs ate the [human] food. You put the salt somewhere, they eat it… but now we have this hutch, we feel safe.” –Mother*

Some participants reported wanting to construct additional hutches in their homes for other animals. A participant who placed only her guinea pigs in the animal hutch said she wanted another hutch for her rabbits, so that the animals would each have their own space. Participants said that their neighbors were very curious about the hutches and inquired about having their own.

*“The model is very good because even the others who are from this neighborhood, when they arrive here, and they see it, they say, ‘that it is good!’ and want to know the person who made it.” –Mother*

### Composting Animal Feces Module

As noted above, the need for a separate Care Group module on composting animal feces emerged from Phase 1 piloting of the Hutches for Small Animals Module. Initially, behavioral recommendations about composting were included in the Hutches for Small Animals Module. However, piloting indicated that this was too much information for a single module, and a distinct module on composting was developed.

#### Pilot – Phase II

We were able to base the Composting Animal Feces Module on parts of an existing Care Group module already in use by Food for the Hungry. We introduced a compost pile made of locally available materials. The module encouraged households to sweep up animal feces in the yard, household, and animal hutch, emphasizing that disease-causing microbes could be present in animal feces. Households were asked to pick a one-by-two-meter plot of land on their property to build their compost pile. We recommended that the compost pile be kept away from where children played.

Most households decided to build their compost piles behind their house, as it was an area rarely used for other purposes. Fences were built around the pile to keep children from touching the compost or falling into the pit. First, sticks and sugar cane stalks were placed down to encourage air circulation at the bottom of the pile. Dried nesting materials, called “brown materials”, were placed on top of the sticks, including dried weeds, mulch, and crop residue. “Green materials”, such as animal excrement, kitchen scraps (waste), and soiled fodder were added next. To hold moisture and add micro-organisms that break down materials, a layer of soil was added on top. This process was repeated until the pile was about a meter high. The module advised households to keep the compost pile moist, to add water to it as needed and to turn the heap with a hoe or shovel every 2-3 weeks. Participants were then told that compost would be ready once it “feels crumbly, smells sweet, and is dark in colour.” Participants were encouraged to use it when planting seeds and to sprinkle it around plants to help them grow.

Participants liked the compost pile because it gave them a place to put animal feces and household waste. Before having a compost pile, one participant said that she had a difficult time keeping her house clean because every time she would sweep and put excrement in a pile, the chickens would gather around the pile and make a mess.

*“I had a serious problem when I swept, it bothers me - you sweep, and you don’t know where to put the [waste]. If you pile it somewhere, the chickens come and scatter it. I used to do this, if I sweep, for example, in the kitchen, I pile the waste in bags in one place, but as we built this compost I already feel free because I sweep, I collect the waste in a basket and I dump it into the compost. I have become comfortable because of the compost.” –Mother*

Participants with the compost piles said they noticed less animal feces and household waste on the household compound, and said that having a place to put animal feces and waste protected them against the spread of disease.

*“[The compost pile] saves us from microbes and dirty things (animal feces and household waste) because we pile all this in the compost and it will no longer spread on the ground, this saves us also from diseases.” –Mother*

Participants also mentioned that the compost pile was good for keeping children away from animal feces and household waste. Children were not able to play with the feces in the compost pile.

*“It is a good thing, because the children won’t be able to play [in the compost pile] with this waste because it’s kept in one place.” –Mother*

Most participants said the compost pile was easy to use and that they liked using the fertilizer for their fields. One participant mentioned that composting was better for the growth of their crops than throwing excrement in the field directly.

*“Today I start to see that those who cultivate with compost benefit a lot from it compared to those who throw it directly in the field. There is a difference.”–Mother*

However, some participants found it difficult to turn the compost heap. Participants also reported that they forgot or did not have the time to turn the compost pile.

Others stated that they did not want to throw animal feces into the compost pile, saying that it took too long to compost feces, and less time to compost herbs and dried materials alone. Therefore, they preferred to compost only kitchen waste.

## Discussion

This research helped to identify existing practices and barriers and facilitators related to Baby WASH behaviors in households with young children in rural DRC, and informed the development of acceptable and feasible Care Group modules to target exposure to fecal pathogens for susceptible pediatric populations.

Formative research informed the development of six Baby WASH Care Group modules for the REDUCE program. This research included semi-structured interviews and focus group discussions with multiple types of caregivers of young children, community leaders, and health workers. This approach allowed for a greater understanding of the community and household context, and the multiple factors likely to influence our targeted Baby WASH behaviors. Findings from exploratory formative research were carefully integrated into Care Group modules, an important step in intervention development that recognizes that what influences behavior change may vary in different settings [20]. This process of integration was facilitated by organizing findings by the multiple dimensions of the IBM-WASH framework, which helped us to identify which determinants could best be addressed by our intervention [20]. Taking a contextually relevant, theory-driven approach to WASH intervention development is a highly underutilized approach despite evidence that it is more likely to result in successful interventions [20, 25–27]. Initial module development was followed by a two-part, iterative pilot, which allowed us to obtain feedback on behavioral recommendations and enabling technology, and make modifications to the intervention as needed.

Caregivers were aware that their children may mouth soil, dirty objects, or feces while playing in the yard, and recognized this behavior as a source of intestinal worms. However, household responsibilities, often outside the home, limited adult caregivers’ ability to intervene on unsafe mouthing behaviors. Similar to other settings, we found that young children are often left in the care of older siblings, or alone [28]. In our study, older siblings sometimes fed younger children play-foods made from mud, like “fufu”, which resulted in children consuming soil. Based on these findings, we included songs describing the risk of mouthing dirt and dirty things in the Protecting Children from Dangers in the Dirt Module in order to make intervention content accessible to children as well as adults. We also included a narrative illustration about a child who ate mud fufu and became sick the next day.

Two challenges impeding safe child feces disposal, safe child mouthing, and separation of children from animals emerged across all formative research activities: limited resources and competing household priorities. As a result, only enabling technology that was low-cost or free, and locally available, was considered for this intervention. In addition, behavioral recommendations that could be integrated into the existing routines of caregivers were prioritized. As one example, we promoted a low-cost, locally available playmat to place young children on while they play in order to limit their contact with dirty floors or soil. This mat was portable and could be brought with caregivers to the field, as demonstrated by pilot study participants. Other studies have explored commercial or community-built play yards to prevent fecal exposure through mouthing behaviors [29, 30]. Play yards were not considered for this study because caregivers would need to be present in the household to supervise children in the play yard, which was not feasible for our participants, and because of cost. Future studies are needed that evaluate the use of protected child play spaces that are built using locally sourced materials based on community recommendations.

In our study, the practice of removing child feces with a hoe and disposing in the latrine was common. However, as demonstrated in multiple settings, infant feces were seen as having fewer germs compared to other human feces [28, 31, 32]. In response, we included information about germs in all feces, including the feces of breastfed infants, in our Safe Child Feces Disposal Module. We also recommended that all diaper wash water be disposed of in the latrine rather than in the field or yard, as was the practice.

Through this study, we were able to design interventions to target novel exposure routes to fecal pathogens not targeted in recent large-scale WASH interventions, specifically, separation of animals and their excreta from young children [33]. This research responds to calls for the development of interventions to limit young children’s exposure to animal feces in the domestic setting [10, 15, 34]. In our study, households kept small animals, including guinea pigs, rabbits, and poultry, loose inside the kitchen or sleeping space. Constructing hutches for small animals may serve to limit children’s exposure to animal feces. In our setting, participants refused to keep small animals outside of the living space due to fears for animal safety. Hutches provide an opportunity to limit direct mouthing of animal feces, a benefit that was reported among our pilot participants. Future studies are needed to evaluate small hutches for animals constructed in the kitchen or sleeping space as a means to reduce young children’s exposure to animal feces.

The iterative approach we took for intervention development was a valuable step in our intervention design, allowing us to identify technological challenges faced by pilot households and to refine the intervention accordingly [25, 35]. For example, feedback from participants about disposal of animal feces prompted the creation of a new module on safely collecting and composting  animal feces after it became clear that animal feces were required for crop productivity and contributed to participants’ livelihoods. In the absence of formative research, recommendations applicable to other settings to dispose of animal feces in the latrine may have been included [36]. Composting animal feces may offer an opportunity for both resource and risk management [37].

These REDUCE Baby WASH Care Group modules have been delivered to over 1 million people in South Kivu and Tanganyika provinces of DRC. Although the REDUCE Baby WASH Care Group modules were developed for rural DRC, the behavioral recommendations, communication materials, and other module content could be adapted for delivery in other settings with further formative research.

This study had important strengths. One was the 24-month duration of the formative research conducted. Second was the iterative nature of the research that allowed us to explore new issues, like compost, that arose as interventions were being developed.

This study has some limitations. First, we did not include government stakeholders in the interview process. They can provide valuable insights on intervention approaches that could be scaled across DRC, and should be included in future studies. Second, we provided enabling technologies to the households for free. Future studies should explore market value for items and assess if households would be willing to pay for them.

## Conclusions

Exposure routes for infants and young children to fecal pathogens in the environment, such as child mouthing behaviors, contact with animals, and fecal contamination on fomites, are often overlooked in WASH interventions. This research took a theory-driven and evidence-based approach to develop acceptable and feasible Baby WASH Care Group modules to promote safe child feces disposal, safe child mouthing, and separation of children from animals. Findings that resource and time constraints were considerable barriers to the performance of positive WASH behaviors in South Kivu prompted a focus on inexpensive interventions that minimized additional demands on the time of  caregivers. Formative research also allowed behavioral recommendations to be tailored to the context. The developed REDUCE Baby WASH Care Group modules are currently being rolled out to over 1,000,000 beneficiaries . Future studies are needed to determine the effectiveness of this intervention in reducing pediatric diarrhea and improving child growth.

## Supplementary Information

**Additional file 1.** Exit Interview Guide.

**Additional file 2.** Handwashing with Soap and Water Treatment Module Appendix.

**Additional file 3: Supplementary Table 3a.** Supporting Quotes for the Design of the Protecting Children from Dangers in the Dirt Care Group Module and Enabling Technology, and IBM-WASH Factors. **Supplementary Table 3b.** Supporting Quotes for the Design of the Safe Disposal of Child Feces Care Group Module and Enabling Technology, and IBM-WASH Factors. **Supplementary Table 3c.** Supporting Quotes for the Design of the Living with Small Animals Care Group Module and Enabling Technology, and IBM-WASH Factors. **Supplementary Table 3d.** Supporting Quotes for the Design of the Composting Care Group Module and Enabling Technology, and IBM-WASH Factors. **Supplementary Table 3e.** Supporting Quotes for Cross Cutting Themes of Limited Resources and Competing Priorities, and IBM-WASH Factors.

## Data Availability

The datasets generated and/or analyzed during the current study are not publicly available due to identifiable content but are available from the corresponding author on reasonable request.

## Abbreviations

WASH: Water, sanitation, and hygiene
REDUCE: Reducing Enteropathy, Diarrhea, Undernutrition, and Contamination in the Environment
DRC: Democratic Republic of the Congo
DFSA: Development Food Security Activity
Baby WASH: WASH interventions for young children
IRB: Institutional Review Board
IBM-WASH: Integrated Behavioral Model for Water, Sanitation and Hygiene

## References

[1] Amouzou A, Velez LC, Tarekegn H, Young M. One is too many: ending child deaths from pneumonia and diarrhea: New York: UNICEF; 2016. p. 5–23.

[2] Guerrant RL, Oriá RB, Moore SR, Oriá MO, Lima AA (2008). Malnutrition as an enteric infectious disease with long-term effects on child development. Nutr Rev.

[3] Lin A, Arnold BF, Afreen S, Goto R, Huda T, Haque R, Raqib R, Unicomb L, Ahmed T, Colford JM, Luby SP (2013). Household environmental conditions are associated with enteropathy and impaired growth in rural Bangladesh. Am J Trop Med Hyg.

[4] George CM, Burrowes V, Perin J, Oldja L, Biswas S, Sack D, Ahmed S, Haque R, Bhuiyan NA, Parvin T, Bhuyian SI (2018). Enteric infections in young children are associated with environmental enteropathy and impaired growth. Tropical Med Int Health.

[5] UNICEF (2018). Estimates of child cause of death, Diarrhoea.

[6] Ministère du Plan et Suivi de la Mise en œuvre de la Révolution de la Modernité (MPSMRM), Ministère de la Santé Publique (MSP) et ICF International. Enquête Démographique et de Santé en République Démocratique du Congo 2013-2014. Rockville: MSP and ICF International; 2014. https://dhsprogram.com/pubs/pdf/FR300/FR300.pdf

[7] Luby SP, Rahman M, Arnold BF, Unicomb L, Ashraf S, Winch PJ (2018). Effects of water quality, sanitation, handwashing, and nutritional interventions on diarrhoea and child growth in rural Bangladesh: a cluster randomised controlled trial. Lancet Glob Health.

[8] Null C, Stewart CP, Pickering AJ, Dentz HN, Arnold BF, Arnold CD (2018). Effects of water quality, sanitation, handwashing, and nutritional interventions on diarrhoea and child growth in rural Kenya: a cluster-randomised controlled trial. Lancet Glob Health.

[9] Humphrey JH, Mbuya MN, Ntozini R, Moulton LH, Stoltzfus RJ, Tavengwa NV (2019). Independent and combined effects of improved water, sanitation, and hygiene, and improved complementary feeding, on child stunting and anaemia in rural Zimbabwe: a cluster-randomised trial. Lancet Glob Health.

[10] Budge S, Parker AH, Hutchings PT, Garbutt C (2019). Environmental enteric dysfunction and child stunting. Nutr Rev.

[11] Morita T, Perin J, Oldja L, Biswas S, Sack RB, Ahmed S (2020). Mouthing of soil contaminated objects is associated with environmental Enteropathy in Young children. Tropical Med Int Health.

[12] Ngure FM, Humphrey JH, Mbuya MN, Majo F, Mutasa K, Govha M et al. Formative research on hygiene behaviors and geophagy among infants and young children and implications of exposure to fecal bacteria. Am J Trop Med Hyg. 2013 89(4):709–716. doi: 10.4269/ajtmh.12-0568. Epub 2013 Sep 3. PMID: 24002485; PMCID: PMC3795101.. 10.4269/ajtmh.12-0568 PMC3795101 24002485

[13] George CM, Oldja L, Biswas S, Perin J, Lee GO, Kosek M (2015). Geophagy is associated with environmental Enteropathy and stunting in children in rural Bangladesh. Am J Trop Med Hyg.

[14] George CM, Oldja L, Biswas SK, Perin J, Lee GO, Ahmed S (2015). Fecal markers of environmental Enteropathy are associated with animal exposure and caregiver hygiene in Bangladesh. Am J Trop Med Hyg.

[15] George CM, Cirhuza LB, Kuhl J, Williams C, Cogmianese N, Thomas E, et al. Child mouthing of Feces and fomites and animal contact are associated with Diarrhea and impaired growth among Young children in the Democratic Republic of the Congo: a prospective cohort study. J Pediatr. 2020. 10.1016/j.jpeds.2020.09.013. 10.1016/j.jpeds.2020.09.013 32918918

[16] Perry H, Sivan O, Bowman G, Casazza L, Edward A, Hansen K, Morrow M. Averting childhood deaths in resource-constrained settings through engagement with the community: an example from Cambodia. Essentials Community Health. 2010:169–74.

[17] Laughlin M (2010). The care group difference: a guide to mobilizing community-based volunteer health educators (2^nd^ edition).

[18] Hanold M, Wetzel C, Davis T (2014). Caregroups: a training manual for program design and implementation.

[19] George CM, Vignola E, Ricca J, Davis T, Perin J, Tam Y, Perry H (2015). Evaluation of the effectiveness of care groups in expanding population coverage of key child survival interventions and reducing Under-5 mortality: a comparative analysis using the lives saved tool (LiST). BMC Public Health.

[20] Dreibelbis R, Winch PJ, Leontsini E, Hulland KRS, Ram PK, Unicomb L, Luby SP (2013). The integrated behavioural model for water, sanitation, and hygiene: a systematic review of behavioural models and a framework for designing and evaluating behaviour change interventions in infrastructure-restricted settings. BMC Public Health.

[21] Morita T, Perin J, Oldja L, Biswas S, Sack RB, Ahmed S et al. Childhood Mouthing of Contaminated Objects is Associated with Environmental Enteropathy. Tropical Med Int Health. 2017. PMID: 28319300 10.1111/tmi.12869 28319300

[22] George CM, Oldja L, Biswas S, Perin J, Lee GO, Kosek M, Sack RB et al. Geophagy is Associated with Environmental Enteropathy and Stunting in Children in Rural Bangladesh. Am J Trop Med Hyg. 2015;92(6):1117-1^23. PMID: 2591821 10.4269/ajtmh.14-0672 PMC4458812 25918214

[23] Davis E, Cumming O, Aseyo RE, Muganda DN, Baker KK, Mumma J, Dreibelbis R (2018). Oral contact events and caregiver hand hygiene: implications for Fecal-Oral exposure to enteric pathogens among infants 3–9 months living in informal, Peri-urban communities in Kisumu, Kenya. Int J Environ Res Public Health.

[24] Yeasmin F, Sultana F, Unicomb L (2019). Piloting a shared source water treatment intervention among elementary schools in Bangladesh. Am J Trop Med Hyg.

[25] Jacob Arriola KR, Ellis A, Webb-Girard A, Ogutu EA, McClintic E, Caruso B, Freeman MC. Designing integrated interventions to improve nutrition and WASH behaviors in Kenya. Pilot Feasib Stud. 2020;6(10). 10.1186/s40814-020-0555-x. 10.1186/s40814-020-0555-x PMC6998333 32042436

[26] Briscoe C, Aboud F. Behaviour change communication targeting four health behaviours in developing countries: a review of change techniques. Soc Sci Med 2012 Aug;75(4):612–621. doi: 10.1016/j.socscimed.2012.03.016. Epub 2012 Apr 7. PMID: 22541798. 10.1016/j.socscimed.2012.03.016 22541798

[27] Inauen J, Mosler HJ (2014). Developing and testing theory-based and evidence-based interventions to promote switching to arsenic-safe wells in Bangladesh. J Health Psychol.

[28] Ellis A, McClintic EE, Awino EO, Caruso BA, Arriola KRJ, Ventura SG et al. Practices and Perspectives on Latrine Use, Child Feces Disposal, and Clean Play Environments in Western Kenya. Am J Trop Med Hyg. 2020;102(5):1094–1103. Doi: 10.4269/ajtmh.19-0389. PMID: 32124727; PMCID: PMC7204574. 10.4269/ajtmh.19-0389 PMC7204574 32124727

[29] Humphrey JH, Mbuya MNN, Ntozini R, Moulton LH, Stoltzfus RJ, Tavengwa NV et al. Sanitation Hygiene Infant Nutrition Efficacy (SHINE) Trial Team. Independent and combined effects of improved water, sanitation, and hygiene, and improved complementary feeding, on child stunting and anaemia in rural Zimbabwe: a cluster-randomised trial. Lancet Glob Health. 2019;7(1):e132-e147. Doi: 10.1016/S2214-109X(18)30374-7. PMID: 30554749; PMCID: PMC6293965. 10.1016/S2214-109X(18)30374-7 PMC6293965 30554749

[30] Reid B, Seu R, Orgle J, Roy K, Pongolani C, Chileshe M, Fundira D, Stoltzfus R (2018). A community-designed play-yard intervention to prevent microbial ingestion: a baby water, sanitation, and hygiene pilot study in rural Zambia. Am J Trop Med Hyg.

[31] Lawrence JJ, Yeboah-Antwi K, Biemba G, Ram PK, Osbert N, Sabin LL, Hamer DH (2016). Beliefs, Behaviors, and perceptions of community-led Total sanitation and their relation to improved sanitation in rural Zambia. Am J Trop Med Hyg.

[32] Chebet JJ, Kilungo A, Alaofè H, Malebo H, Katani S, Nichter M. Local Perceptions, Cultural Beliefs, Practices and Changing Perspectives of Handling Infant Feces: A Case Study in a Rural Geita District, North-Western Tanzania. Int J Environ Res Public Health. 2020;17(9):3084. doi: 10.3390/ijerph17093084. PMID: 32365476; PMCID: PMC7246464. 10.3390/ijerph17093084 PMC7246464 32365476

[33] Pickering A, Null C, Winch P, Mangwadu G, Arnold BF, Prendergast (2019). The WASH Benefits and SHINE trials: interpretation of WASH intervention effects on linear growth and diarrhoea. Lancet Glob Health.

[34] Penakalapati G, Swarthout J, Delahoy MJ, McAliley L, Wodnik B, Levy K, Freeman MC. Exposure to Animal Feces and Human Health: A Systematic Review and Proposed Research Priorities. Environ Sci Technol. 2017;51(20):11537–11552. doi: 10.1021/acs.est.7b02811. Epub 2017 Oct 9. PMID: 28926696; PMCID: PMC5647569. 10.1021/acs.est.7b02811 PMC5647569 28926696

[35] The Manoff Group (2005). Trials of improved practices (TIPs): giving participants a voice in program design.

[36] Arnold BF, Null C, Luby SP, Unicomb L, Stewart CP, Dewey KG (2013). Cluster-randomised controlled trials of individual and combined water, sanitation, hygiene and nutritional interventions in rural Bangladesh and Kenya: the WASH Benefits study design and rationale. BMJ.

[37] Sabiiti EN (2011). Utilising agricultural waste to enhance food security and conserve the environment. Afr J Food Agric Nutr Dev.

