# Supplementary material for: Formative research for the development of baby water, sanitation, and hygiene interventions for young children in the Democratic Republic of the Congo (REDUCE program)
Source: BMC Public Health. 2021 Mar 1;21:427. doi: 10.1186/s12889-021-10246-5 (PMC7923459; doi:10.1186/s12889-021-10246-5)
Supplement: Supplementary file 1 — Additional file 1. Exit Interview Guide. [file 12889_2021_10246_MOESM1_ESM.docx]

**JOHNS HOPKINS BLOOMBERG SCHOOL OF PUBLIC HEALTH**

**DATA COLLECTION TOOL**

**REDUCE PROGRAM**

**Exit Interview Guide**

Interviewer’s Name: ____________________________________________________

Village’s Name: _______________________________________________________

Date: ____/____/____ Start time: ___:___AM/PM End time: ___:___AM/PM

DD MM YY

PARTICIPANT ID: ______________________________________________________

**PROMPT**

Hello, and thank you for agreeing to speak with me today. Your thoughts on this subject will be very helpful to us. There are no right or wrong answers to these questions, please answer as you like. We have a recording device to help us remember what you say today, and we will take some notes. Your identity will be confidential.

**Module 1: Dangers in the Dirt**

**1.1** We would like your feedback on the playmats we provided. Let’s discuss:

**1.1a** What was your experience with the playmats?

**1.1b** What about it was easy?

**1.1c** What about it was difficult?

**1.1d** Is there something you want more information on?

**1.1e** Is there something you would like to change about the playmat?

**1.1f** Who in your household participated in this behavior? Why?

**1.1g** Who in your household did not participate in this behavior? Why?

**1.1h** When was your child most often on the playmat?

**1.1i** When your child was on the playmat, what did they do most often?

**Module 2: Safe Child Feces Disposal**

**2.1** We would like your feedback on our program on disposing of child feces safely. Let’s discuss:

**2.1a** What was your experience with this program?

**2.1b** What about it was easy?

**2.1c** What about it was difficult?

**2.1d** Is there something you want more information on?

**2.1e** Is there something you would like to change about the recommendation?

**2.1f** Who in your household participated in this behavior? Why?

**2.1g** Who in your household did not participate in this behavior? Why?

**Module 3: Hutches for Small Animals**

**3.1** We would like your feedback on our program on keeping your child’s play space clean. Let’s discuss:

**3.1a** What was your experience with this program?

**3.1b** What about it was easy?

**3.1c** What about it was difficult?

**3.1d** Is there something you want more information on?

**3.1e** Is there something you would like to change about the recommendation?

**3.1f** Who in your household participated in this behavior? Why?

**3.1g** Who in your household did not participate in this behavior? Why?

**Module 4: Compost Model**

**4.1** We would like your feedback on our composting program.

Let’s discuss:

**4.1a** What was your experience with this program?

**4.1b** What about it was easy?

**4.1c** What about it was difficult?

**4.1d** Is there something you want more information on?

**4.1e** Is there something you would like to change about the recommendation?

**4.1f** Who in your household participated in this behavior? Why?

**4.1g** Who in your household did not participate in this behavior? Why?

**Module 5: Handwashing with Soap Module**

**5.1** We would like your feedback on our program on handwashing with soap/chlorine at key times. Let’s discuss:

**5.1a** What was your experience with this program?

**5.1b** What about it was easy?

**5.1c** What about it was difficult?

**5.1d** Is there something you want more information on?

**5.1e** Is there something you would like to change about the recommendation?

**5.1f** Who in your household participated in this behavior? Why?

**5.1g** Who in your household did not participate in this behavior? Why?

**Module 6: Water Treatment Module**

**6.1** We would like your feedback on our program on water treatment. Let’s discuss:

**6.1a** What was your experience with this program?

**6.1b** What about it was easy?

**6.1c** What about it was difficult?

**6.1d** Is there something you want more information on?

**6.1e** Is there something you would like to change about the recommendation?

**6.1f** Who in your household participated in this behavior? Why?

**6.1g** Who in your household did not participate in this behavior? Why?
