# Supplementary material for: Formative research for the development of baby water, sanitation, and hygiene interventions for young children in the Democratic Republic of the Congo (REDUCE program)
Source: BMC Public Health. 2021 Mar 1;21:427. doi: 10.1186/s12889-021-10246-5 (PMC7923459; doi:10.1186/s12889-021-10246-5)
Supplement: Supplementary file 2 — Additional file 2. Handwashing with Soap and Water Treatment Module Appendix. [file 12889_2021_10246_MOESM2_ESM.docx]

**Supplementary File 2**

**Handwashing with Soap and Water Treatment Module Appendix**

## Handwashing with Soap Module

### Exploratory Formative Research

All caregivers reported having to collect water from a source outside their household compound, and none reported having a designated vessel to store handwashing water. Some caregivers stated that the lack of water in the household prevented them from washing their hands as often as they might want to:

“I wish we were closer to water because sometimes you need to wash your hands, but there is no water. Sometimes you find a sweet potato in the house, and you want to eat it, but there's no water, so you ask yourself: "Will I first go to look for water [before] I eat my sweet potato - because there isn't any water here? I will just eat it as I am [without handwashing].” – Mother

Some participants mentioned using soap for handwashing, but many noted that if they ran out of soap, they might not be able to afford to replace it and would simply wash their hands with water. Caregivers reported washing their hands by dipping them in a basin filled with water. Designated handwashing stations and soap were both perceived as expensive:

“There are those who build nice houses and build a place for washing hands, and others take tables and put a bucket with a tap and a basin below…they say it costs 10 dollars.” – Mother

When soap was purchased, caregivers reported using it for household chores, such as laundry or dishwashing, more often than handwashing. Caregivers reported washing their hands at different key times, including: before preparing food, before eating, after returning from working in the field, after toileting, and after cleaning a child’s bottom. However, few reported washing their hands with soap at *all* these critical points. The majority of participants reported consistently washing their hands first thing in the morning.

In addition to the lack of water and soap, forgetting to wash hands, or adults being absent from the home, were cited as barriers to handwashing with soap among children.

“This is the problem we have with keeping a child's hands clean—we can't stay with him all the time wherever he is. You will go to the field and leave the child to eat and the child will rush to eat without washing his hands and eat only like that.” – Village leader

### Pilot - Phase I

Participants had told us that water was rarely put aside for handwashing, and that if they did wash their hands, they did so by dipping hands in a shared basin—a method that does not allow hands to be properly cleaned. To address these issues and facilitate effective handwashing with soap behavior, two models of handwashing stations were proposed in Phase I of piloting: a tippy tap (1 USD) and a plastic bucket with an attached tap (8 USD). Both items are locally available.

Two inexpensive soap options were provided to all families: locally manufactured bar soap (0.1 USD), and a soapy water bottle (powdered detergent and water in a used 500 mL water bottle). A sachet of detergent powder costs 0.2 USD and can make approximately 3 bottles of soapy water). To reduce the use of provided soap for purposes other than handwashing, both soap options were attached to the hand washing stations with a string.

The accompanying flipbook provided pictorial instructions on how to make soapy water and how to put soap on a string. The low-cost of these options in comparison with imported handwashing soap (1 USD), which was deemed too expensive by pilot participants, was also highlighted. The flipbook built on disgust expressed regarding eating feces, showing the many ways feces might be ingested if hands were not washed. The flipbook also included pictures depicting the dangers of handwashing by dipping everyone’s hands into a single basin instead of rinsing hands with running water. It also showed critical times for handwashing with soap by explaining that, in the same way everyone washes their hands in the morning, hands must also be washed before eating and after touching feces. The flipbook showed photos of both adults *and* children practicing proper handwashing with soap technique.

Participants responded positively to both the tippy tap and the plastic bucket with the tap, and recipients of both technologies reported using them at key moments for handwashing with soap. Some participants who received the bucket and faucet combination expressed concern that it would be stolen, and stored the station inside the house when they worked away from home. Some participants also reported that the tap on the plastic bucket was leaking:

“When I go to the field, I leave [the plastic bucket with tap] here (in the house)… Because someone might steal it. And when I finish in the field, I put it outside again.” - Mother

One household who received the tippy tap suggested that neighbors wanted one of their own. Research staff observed that some households living near pilot households constructed their own tippy taps based on our recommended design. As one participant said:

“The neighbors have appreciated this [tippy tap] … One of my neighbors who asked me to build one at her place but I have not yet had time. You just need a 5-liter jerry can, and that is about 500fc and also buy soap at 300fc.”- Mother

*Intervention Refinement for Phase II Piloting*

The lower cost of the tippy tap, and the concerns of theft and leakage with the plastic bucket with a faucet, led us to select the tippy tap for Phase II Piloting. To make behavioral recommendations more accessible to children and to encourage handwashing among children while parents were absent, the module for Phase II Piloting included two songs—one which describes proper handwashing techniques and the other which explains key moments for handwashing with soap.

*Phase II Piloting*

In the interviews completed after the second phase of the pilot, participants reported that the tippy tap was easy to use and that it was something that they and others in their community perceived as valuable:

“It’s very important [the tippy tap]. It makes it easier for everyone to wash their hands.” – Mother

“It’s a very important place it facilitates hand washing for everyone, there are many who also want to have a place like this at home in my village.” - Father

One participant said that they liked the tippy tap enough to consider building a second one outside the front of the house for guests to use when they arrived:

“I could build a place for hand washing besides [the one you gave me]. I’d like to put another hand washing device in front of my house so that the people who enter the house wash their hands there directly instead of going to [the tippy tap beside] the toilet.” – Father

This same participant noted that the materials used to the construct the tippy tap were simple and accessible, and it would therefore be easy for them to construct one of their own.

Participants expressed differing preferences for the type of soap used with the tippy tap. Some participants preferred the bar soap because children played with the soapy water and wasted it, while others preferred the soapy water because children used it frequently:

“I liked the hard soap more (bar soap) than the liquid soap (soapy water). The children play with the liquid soap and pour it all over the floor.” - Mother

“I liked the [powdered] soap – that was good because even the children came to pour it and wash with it.” Mother

It was noted by one participant that the tippy tap and accompanying soap allowed for a change in handwashing behavior.

“It became a habit for me, during the day I wash my hands all the time – if I touch dirt, I directly have to wash my hands with soap.” - Father

## Water Treatment Module

Participants stated that they collect drinking water from water sources outside their homes, and may travel up to thirty minutes to get to a water source. Many participants reported storing water in the same containers in which it was collected, and using the same water for all activities.

“We keep it [the water] in the jerry can where it is collected. It is the same water that we drink … and use for other work.” - Mother

Many participants described the value of boiling water, even mentioning that it killed microbes; however, few people reported actually boiling water. Various reasons were given for not boiling water including lack of time. Some participants also mentioned forgetting to boil water in advance of wanting to drink it:

“I might also want to [boil water]… because [then] it does not have microbes, but often we forget to do it and we lack the time to do it.” - Mother

Other barriers to boiling water included having numerous other household tasks, “laziness,” and challenges finding or purchasing fuel or firewood.

Some participants also indicated that not having a place to store the water after boiling it disincentivized boiling the water:

“I can boil it, but I won't be able to find a place to put it - if I put it in a small jerry can or somewhere many things can happen to it.” - Mother

The most commonly used term to describe water sources is “the source” and in this region, this is commonly either a streams or s spring with a cement structure with a few pipes coming out. Some participants also expressed a belief that water coming from a source was clean and did not need to be treated. Participants said they’d been drinking from their water source for a long time, with no treatment, without getting sick:

“Here we believe that the water coming from the source is the best and it isn't necessary to add any treatment to drink it because it can't have any microbes.” - Mother

When participants were asked about whether they had ever seen water being treated with chlorine or tablets, some suggested that the water was already being treated at the source. The participants who said this used the word *banatiyaka* meaning “medicine”:

“I've heard them say they there is a medicine they put in the [central] tap, but I've never seen it.” - Mother

Very few participants had seen chlorine tablets for home water treatment, and those who had seen them had observed them in urban centers rather than in rural communities. When presented with the idea of water treatment tablets, some participants thought that if they could find a place to buy tablets, using them would be easier than boiling water, because they would be faster and would not require hunting for firewood.

“It is difficult to find either charcoal or firewood for [boiling]. It can also happen that you do not have time to boil... This is why, for us, these water treatment drugs would be easier to use to treat our drinking water than boiling it.”- Mother

### Phase I Piloting

Based on the findings that drinking water is stored in the same container where it is collected, and concerns that the water could be re-contaminated after it is treated, a jerry can with a plastic tap installed near the bottom to dispense water was given to participating families. The jerry cans were labelled with a sticker showing a person drinking from a cup and the words “Drink the water that is good for your health”; these labels were added to promote drinking water only from this jerry can. Participating families were also given a supply of 30 chlorine tablets for treating their water to address time constraints to boiling water noted during exploratory research.

The flipbook designed to accompany the chlorine and jerry can with tap included instructions for using and storing the chlorine tablets safely. The flipbook showed that water that looks clean can be contaminated, responding to participant suggestions that water coming from the source was clear and uncontaminated. Bottled water was highly valued in the area . As such the flipbook drew comparisons between bottled water and water treated at home with chlorine, noting that both often contain chlorine. The pictorial module also showed that even with the half hour wait period between water treatment with chlorine and consumption, chlorination is faster than treating water by boiling, which requires finding wood for fuel, waiting for water to boil, and then waiting for water to cool.

Participants responded positively to the jerry can with tap and chlorine tablets. Participants said that they used the water from the jerry can with tap whenever they were thirsty, and that even their younger children quickly adapted to drinking treated water only:

“Even the smallest child will no longer accept drinking water that isn’t from this jerry can [with tap] because he already knows the water in this jerry can is for drinking.” - Mother

Participants noted that the chlorine changed the smell and taste of the water, but said this did not dissuade them from drinking it. They reported that the chlorine tablets were easy to use and they appreciated that the water was ready to drink in a short amount of time:

“There is nothing I see that is difficult because I only have to put in the tablet and we can begin to drink it after thirty minutes.” - Mother

Participants said they would be willing to pay for chlorine tablets, but raised concerns about being able to find them locally.

### Intervention Refinement for Phase II Piloting

Noting both that families might not have consistent access to chlorine tablets, and that there were challenges to boiling water, for Phase II piloting we recommended that households save some of the boiled water made when preparing fufu to give to young children in the household for drinking. It was also recommended that households cover their drinking water once it is treated to reduce re-contamination. In addition, to reinforce the teachings of this lesson, a narrative illustration showing that untreated water can make you sick, and a song with the same statment, were included to target older children in the home.

### Phase II Piloting

Interviews conducted after Phase II piloting elicited similar positive responses. Respondents said the chlorine tablets and jerry can were easy to use. As was the case with the other technologies, people reported that their neighbors were curious about the jerry can with tap and wondered how they might be given one as well:

“It is a good thing - it allows us to keep our water clean, besides everyone in my community wants to have it, too.” – Mother

One participant said that her child no longer has diarrhea after using the chlorine tablet:

“I am very happy because since we started drinking this water in my house, my child no longer suffers from diarrhea… my smallest child was suffering a lot from diarrhea and I was wondering if this is an epidemic because it is often said that when a child starts to crawl, he gets diarrhea. But since we started using this treated water, I have never seen my child have diarrhea, so it's a good program and I'm happy.” – Mother

When participants were asked about the smell of chlorine-treated water, some said they were not concerned because they knew that there were no microbes in the water:

“[Our neighbor said] this water is different than the one we consume directly from the source; it smells like medicine. It’s this medicine that neutralizes the microbes! [Our neighbor] understands …that the source from which we draw water is infected.”—Father

Others said that some members of their family did not like the smell of the water:

“There are people who say the water has a bad smell like the water in Bukavu, it’s not good, it smells like medicine…my husband’s younger sister refused to drink this water, she said it stinks like medicine.”

Participants mentioned that chlorine tablets were more convenient than boiling due to the volume of water that could be treated, and the faster speed of treatment.

“With a tablet you easily have 20L [of clean water]. And at times we are impatient to wait for the boiling [water] and while the water is cooling we are thirsty, so we feel obliged to consume the water untreated. But this other method [chlorine tablets] only takes thirty minutes to have pure water.”—Mother

# **Supplementary Table 1a. Design of the Handwashing with Soap Care Group Module and Enabling Technology, Organized by IBM-WASH Dimension**

| **Dimension of IBM-WASH** | **Implications for intervention design** |
| --- | --- |
| *Contextual dimension* |  |
| Roles and responsibilities: Because adult caregivers often work away from the home, infants and toddlers may be left in the care of school-age children (4-12). | Songs describing the steps for handwashing with soap and key times for handwashing with soap developed to make behavioral recommendations about handwashing accessible to school-age children. |
| Barriers to repetition of behavior: Soap is used for other purposes (laundry, dishes) and may not be consistently available for handwashing. | Soap provided to households, with a string that attached it to a handwashing station to avoid use of that soap for other household purposes. Soapy water provided as a low cost alternative to bar soap. |
| *Psychosocial dimension* |  |
| Remembering: Participants do not always remember to wash their hands at key times. | Pictorial instructions included in module suggesting the handwashing station or tippy tap be placed near the latrine to support household members remembering to wash hands after toileting. |
| Existing habits: Most people report washing their hands in the morning when they wake up. | Pictorial instructions included in module mentioning this morning handwashing practice and connecting it to the other key times for handwashing. |
| *Technology dimension* |  |
| Access: There is no piped water into homes in these communities. | Handwashing station or tippy tap provided to households as an enabling technology to allow water to flow over hands and facilitate handwashing. |
| Existing habits: Hands are washed by dipping them in a shared basin. |  |
| Perceived cost: Soap and handwashing stations perceived as expensive. | Tippy tap selected as final enabling technology due to lower cost. Soap options provided were low cost. |

# **Supplementary Table 1b. Design of the Water Treatment Care Group Module and enabling technology, Organized by IBM-WASH Dimension**

| **Dimension of IBM-WASH** | **Implications for intervention design** |
| --- | --- |
| *Contextual dimension* |  |
| Roles and responsibilities: Female caregivers have many household chores, and collecting fuel for boiling and actually boiling water is time consuming. | Water treatment tablets provided to households because they are less labor intensive than boiling water, and do not require fuel collecting or other related costs. |
| Household resources: Fuel may be expensive, or challenging and time-intensive to find |  |
| *Psychosocial dimension* |  |
| Perceived susceptibility: Individuals reported drinking water without treatment and not getting sick. | Narrative illustration included in module highlighting that children need extra care to stay healthy and can get sick from drinking untreated water. |
| Perceived benefits/outcome expectations: One mother who boiled her water consistently said she noticed her children were sick less after she boiled water. | Narrative illustration included in module featuring a story of a mother who gave her child boiled water and the child being healthier than before. |
| Existing habits: Households collect and store water in the same jerry can, and use that water for all purposes without treatment. | Participants were provided with a designated jerry can for safe drinking water storage with an installed tap to prevent recontamination |
| *Technology dimension* |  |
| Access/availability: Most participants had never seen chlorine tables before. | Pictorial instructions included in module providing information about chlorine tablets, and how they are faster than boiling water. |

# **Supplementary Table 2a. Supporting Quotes for the Design of the Handwashing with Soap Care Group Module and Enabling technology, and IBM-WASH Factors**

| Quote | IBM-WASH Factor |
| --- | --- |
| I wish we were closer to water because it happens that you need to wash your hands, but there is no water. Sometimes you find a sweet potato in the house, and you're going to eat it but there's no water, so you ask yourself "Will I first go to look for water to eat my sweet potato because there isn't any water here? I can eat it only like that [without handwashing]." | *Built and physical environment*  *Access*  *Barriers to repetition of behavior* |
| If you tell me you'd like to wash your hands I will bring you a small basin designated for handwashing and a cup and I will pour the water for you to wash your hands. | *Existing habits/practices* |
| There are some people who have handwashing stations in their homes but not everyone. There are those who build nice houses and build a place for washing hands and others take tables and put a bucket with a tap and a basin below…they say it costs 10 dollars. | *Household resources*  *Cost* |
| You take your water and you put it in a basin with soap and you wash and you make your hands clean and that's all, like that! | *Existing habits/practices* |
| Here, we don't have laundry soap that is different from handwashing soap, that’s why when we buy soap for washing clothes and we finish washing clothes the soap that remains can be used for washing hands. | *Household resources*  *Barriers to repetition of behavior* |
| After some time, it can happen again that there are two weeks or a month without having soap, then you have a problem but for an adult, they’re going to begin to use ashes but a child can't. | *Barriers to repetition of behavior* |
| You can see a person passing their whole day without washing their body, but a person can't get up in the morning without first washing their hands. | *Existing habits/practices* |
| At the time we want to eat, when we leave the field, when we want to breastfeed a child, we wash our hands. After you’ve worked if you want to prepare food you have to wash your hands … | *Roles and responsibilities* |
| We have difficulty obtaining soap. If you sell bananas for 500fc, you pay 200fc for soap and this soap is going to be used for laundry but the "wilt" has already ravaged all the banana plantations - so we have a problem of having soap here because the banana plantations are dry. | *Cost*  *Household resources* |
| Sometimes… soap is not available in a household, but also sometimes you don't have the means to buy it. | *Perceived cost*  *Barriers to repetition of behavior* |
| This is the problem we have with keeping a child's hands clean—we can't stay with him all the time wherever he is. You will go to the field and leave the child to eat and the child will rush to eat without washing his hands and eat only like that. These microbes [from his hands] he puts them in his stomach. | *Roles and responsibilities* |
| There also are older people as well – [a woman] comes and rushes to the food without remembering that she gave a lot of greetings along the way. She will not remember that she came from the toilet or the field without washing and she will jump on food like a chicken. | *Remembering* |

# **Supplementary Table 2b. Supporting Quotes for the Design of the Water Treatment Care Group Module and Enabling technology, and IBM-WASH Factors**

| Quote | IBM-WASH Factor |
| --- | --- |
| Interviewer: From here to the source, how much time does it take? Participant: It can take maybe 30 minutes. | *Physical environment*  *Access* |
| We keep it in the jerry can where it is collected. It is the same water that we drink … and use for other work. | *Existing habits/practices* |
| I can boil it, but I won't be able to find a place to put it - if I put it in a small jerry can or somewhere, many things can happen to it | *Household resources* |
| [Chlorine] tablets?... No, I've never heard of them. | *Access*  *Availability* |
| I also want to [boil water] because we say that [boiled water] is good because [then] it does not have microbes, but often we forget to do it and we lack the time to do it. | *Knowledge*  *Perceived benefits*  *Remembering*  *Roles and responsibilities* |
| Here, for us to eat, everything comes from the field and when we come back, we're busy with other tasks in the house such as drawing water, washing utensils and preparing a meal for the family. In the end, you find that it is too late -there are other household activities that have not been done like boiling water for the family. | *Roles and responsibilities* |
| I've heard them say they there is a treatment they put in the [central] tap, but I've never seen it. | *Perceived prevalence water treatment* |
| Boiling… it is difficult to find firewood for this purpose. It may also happen that there is no time to boil water. | *Household resources*  *Availability*  *Access*  *Roles and responsibilities* |
