# Supplementary material for: Formative research for the development of baby water, sanitation, and hygiene interventions for young children in the Democratic Republic of the Congo (REDUCE program)
Source: BMC Public Health. 2021 Mar 1;21:427. doi: 10.1186/s12889-021-10246-5 (PMC7923459; doi:10.1186/s12889-021-10246-5)
Supplement: Supplementary file 3 — Additional file 3: Supplementary Table 3a. Supporting Quotes for the Design of the Protecting Children from Dangers in the Dirt Care Group Module and Enabling Technology, and IBM-WASH Factors. Supplementary Table 3b. Supporting Quotes for the Design of the Safe Disposal of Child Feces Care Group Module and Enabling Technology, and IBM-WASH Factors. Supplementary Table 3c. Supporting Quotes for the Design of the Living with Small Animals Care Group Module and Enabling Technology, and IBM-WASH Factors. Supplementary Table 3d. Supporting Quotes for the Design of the Composting Care Group Module and Enabling Technology, and IBM-WASH Factors. Supplementary Table 3e. Supporting Quotes for Cross Cutting Themes of Limited Resources and Competing Priorities, and IBM-WASH Factors. [file 12889_2021_10246_MOESM3_ESM.docx]

# Supplementary File 2

**Supporting Quotes for the Design of REDUCE Modules**

# Supplementary Table 3a. Supporting Quotes for the Design of the Protecting Children from Dangers in the Dirt Care Group Module and Enabling Technology, and IBM-WASH Factors

| **Quote** | **IBM-WASH Factor** |
| --- | --- |
| In the city, a child can be on a balcony where there is a carpet or mat that the mother has placed there because she's already educated, but here in the village everywhere he's going to crawl is earth only… he's a baby, he can see the mud and play with it. | *Physical environment* |
| You see here, outside, there is the ground, and if there was a clean place ... he wouldn't eat dirt. If we had a tarp, we could sit the infant on the ground and he wouldn't eat dirt anymore. | *Physical environment* |
| Interviewer: Where does your child play during the day when you’re working?  Participant: He plays in the yard. | *Existing habits/practices* |
| It happened that I placed the child in an enclosure but the child did not accept being enclosed that way – he cried and wanted me to take him from that place. | *Concern/Fear* |
| If a parent is present or the other children with whom he plays, the child will not be afraid. | *Concern/Fear* |
| Interviewer: What diseases usually happen during the year? Participant: Diseases like "snakes in the belly" [intestinal worms] and diarrhea | *Knowledge*  *Perceived threat* |
| Interviewer: What kinds of things don't you like them to put in their mouth? Participant: Soil, bottles, dirty clothes … there are things a child puts in their mouth and then it sticks in their intestines and can cause other diseases. | *Dislike*  *Perceived threat* |
| [Children] say they've made fufu with this mud, and sometimes they take branches that fall in the banana plantation and use them like a plate and begin to eat… like the little boy who is there, he eats it and swallows because he doesn't know better. | *Existing habits/practices*  *Knowledge* |
| Little children spend the day in the yard alone because of the bad life we live today. Their mother goes to the field as does their father, and the child isn't supervised because there isn't anyone present to watch him. He can eat whatever he wants and does whatever he wants. | *Existing habits/practices*  *Roles and responsibilities*  *Poverty* |
| Interviewer: And when you go to the field, you leave your two-year-old at the house? Participant: Yes, I leave him with his four-year-old brother. I leave them and I say "I'm leaving you here and I'll come back. Rest and play until the others get back from school." | *Roles and responsibilities* |
| If we were in a place with means, if we could buy the child a toy … that would help him not to touch everything. | *Household resources*  *Access* |
| Here, when a child starts to crawl, he is put on the ground and then he picks up everything he finds and puts in in his mouth. | *Physical environment*  *Existing habits/practices* |
| Interviewer: How do you find it when children eat feces? Participant: It's poison - that's what our parents said. A person can't eat feces and if they eat them, they can't continue to live because they contain a lot of microbes. | *Perceived threat*  *Beliefs* |

# Supplementary Table 3b. Supporting Quotes for the Design of the Safe Disposal of Child Feces Care Group Module and Enabling Technology, and IBM-WASH Factors

| **Quote** | **IBM-WASH Factor** |
| --- | --- |
| The feces of a small child who has already started to eat has many microbes, and an infant who has not yet started to eat all sorts of food does not have too many microbes. | *Perceived threat*  *Beliefs* |
| You find the caregiver is in the house and the child will crawl and defecate. The caregiver isn’t close and doesn’t know, so the child goes and defecates in another place and when you pass the yard, you'll find feces everywhere. | *Roles and responsibilities*  *Existing habits/practices*  *Access* |
| We must remove [the feces] with a hoe and throw it in the toilet and it's our job, we can't not do it. | *Existing habits/practices*  *Compelled to act* |
| We had small toilets (shallow holes in the ground) for the children but the people of Village Assaini told us never again to use these toilets because they were a source of flies which bring microbes everywhere in the area... [to build the toilets] you just dig a little hole and then you put sticks of wood and that's it. [VA] told us they were very bad, so we destroyed them. | *Prior programs/communication*  *Existing habits/practices*  *Knowledge* |
| I throw [the feces] in [the toilet] after I return. I wash his clothes and spread them out… I throw the first wash water in the hole and the second wash I throw in the yard. | *Existing habits/practices* |
| Interviewer: And this water that you used to wash the baby (after defecation), where do you throw it? Participant: In the banana plantation | *Existing habits/practices* |
| Feces have microbes that come from infants and other children who are already eating food. And when you get the idea in your head that it's human excrement, it always makes you nauseated. | *Beliefs*  *Disgust reaction* |

# Supplementary Table 3c. Supporting Quotes for the Design of the Living with Small Animals Care Group Module and Enabling Technology, and IBM-WASH Factors

| **Quote** | **IBM-WASH Factor** |
| --- | --- |
| The ancients lived with their cows without a problem. | *Cultural identity* |
| We've lived together with animals for a longtime, that's how we raise them here. We're already used to it and it doesn't cause a problem. | *Cultural identity*  *Existing habits/practices* |
| It's also to avoid theft. When you have two or three houses, you put the animals you're raising in one house and you stay in another. Two or three days after people understand that nobody is sleeping in the house where the animals are, thieves will visit you and steal… Like me - yesterday I had a chicken stolen from the house because they knew that no one was spending the night there. | *Theft* |
| The rabbit has a lot of attacks and a lot of disturbances and if you let him roam freely all over the house it is not good. [A rabbit] requires a safe place where you feed it and keep it clean. | *Safety of animals* |
| The kitchen is the best place [for rabbits and guinea pigs]… if we put them [outside, it] is very cold and they will die. I once kept guinea pigs [outdoors] and they died – and I even put the rabbits [outside], and they died too, and people began to advise not to keep animals where there is no fire, so I tried putting them in the kitchen and they did not die anymore. | *Safety of animals* |
| Interviewer: And these guinea pigs, where do you keep them?  Participant: The guinea pigs? In the kitchen. | *Existing habits/practices* |
| Interviewer: And this chicken spends the day where? Participant: She circulates around our area. Interviewer: And the night? Participant: She sleeps in the house… in our kitchen. | *Existing habits/practices* |
| The people who spend the night [with their animals] lack the means [to keep them apart] though the person has one side and the animal has the other side…we Africans do not have the means that would allow us to live in a house apart and have another house for animals. | *Household resources* |
| I have guinea pigs but when a child falls ill, like if he's missing blood, I begin to fatten them up [guinea pigs] and he [the child] eats them. | *Benefits* |
| Interviewer: When you see him playing with the guinea pigs like that …  Participant: I don't like it because he might kill [the guinea pig]. | *Safety of animals* |
| A lot of diseases, they are the ones [animals] that cause a lot of diseases…like malaria for example, because animals have a lot of dirt, bacteria come from animals. | *Perceived threat* |
| After touching [cow] dung, we always say it's not dirty because it has come freshly from the stomach of a cow where there is no dirt. | *Perceived threat* |
| [Chicken feces] demand sweeping and throwing in the field because they fertilize our fields. | *Livelihood/crop productivity*  *Existing habits/practices* |

# Supplementary Table 3d. Supporting Quotes for the Design of the Composting Care Group Module and Enabling Technology, and IBM-WASH Factors

| **Quote** | **IBM-WASH Factor** |
| --- | --- |
| I had a serious problem when I swept, it bothers me, you sweep and you don’t know where to put the [waste]. If you pile them somewhere, the chickens come and scatter them. | *Existing habits/practices*  *Household resources* |
| I stock them [animal feces] in one place and after two days or a week I take them to the field. | *Existing habits/practices* |
| I throw [the animal feces] in the onion field … my child eats it [the animal feces], Jesus, what he sees is what he’ll eat …and [the field] is close | *Existing habits/practices* |
| Animals’ excrement is important to us because we use them as fertilizer. | *Benefits* |

# Supplementary Table 3e. Supporting Quotes for Cross Cutting Themes of Limited Resources and Competing Priorities, and IBM-WASH Factors

| **Quote** | **IBM-WASH Factor** |
| --- | --- |
| The big problem is hunger and poverty that make us suffer a lot - there are no sweet potatoes, no bananas, and no cassava. Here, where we are, many have not eaten and do not know if they will eat today. It is because of poverty that brings us a lot of things, there is no money and no harvest. | *Poverty*  *Food security* |
| Sometimes a child can get sick and you leave him at home because you lack the money to treat him… They're not going to give you medicine for nothing. And if you treat him today, tomorrow you can't [get treatment] because you won’t have money. | *Household resources*  *Cost*  *Barriers to treatment* |
| Here, for us to eat, everything comes from the field and when we come back, we're busy with other tasks in the house such as drawing water, washing utensils and preparing a meal for the family. In the end, you find that it is too late -there are other household activities that have not been done like boiling water for the family. | *Division of labor/Roles and responsibilities* |
